# Effects of Lactate Administration on Mitochondrial Respiratory Function in Mouse Skeletal Muscle

**DOI:** 10.3389/fphys.2022.920034

**Published:** 2022-06-30

**Authors:** Kenya Takahashi, Yuki Tamura, Yu Kitaoka, Yutaka Matsunaga, Hideo Hatta

**Affiliations:** ^1^ Department of Sports Sciences, The University of Tokyo, Tokyo, Japan; ^2^ Graduate School of Health and Sport Science, Nippon Sport Science University, Tokyo, Japan; ^3^ Research Institute for Sport Science, Nippon Sport Science University, Tokyo, Japan; ^4^ Department of Human Sciences, Kanagawa University, Yokohama, Japan

**Keywords:** lactate, mitochondria, oxygen consumption rate, supercomplex, skeletal muscle

## Abstract

Recent evidence has shown that mitochondrial respiratory function contributes to exercise performance and metabolic health. Given that lactate is considered a potential signaling molecule that induces mitochondrial adaptations, we tested the hypothesis that lactate would change mitochondrial respiratory function in skeletal muscle. Male ICR mice (8 weeks old) received intraperitoneal injection of PBS or sodium lactate (1 g/kg BW) 5 days a week for 4 weeks. Mitochondria were isolated from freshly excised gastrocnemius muscle using differential centrifugation and were used for all analyses. Lactate administration significantly enhanced pyruvate + malate- and glutamate + malate-induced (complex I-driven) state 3 (maximal/ATP synthesis-coupled) respiration, but not state 2 (basal/proton conductance) respiration. In contrast, lactate administration significantly decreased succinate + rotenone-induced (complex II-driven) state 3 and 2 respiration. No significant differences were observed in malate + octanoyl-l-carnitine-induced state 3 or 2 respiration. The enzymatic activity of complex I was tended to increase and those of complexes I + III and IV were significantly increased after lactate administration. No differences were observed in the activities of complexes II or II + III. Moreover, lactate administration increased the protein content of NDUFS4, a subunit of complex I, but not those of the other components. The present findings suggest that lactate alters mitochondrial respiratory function in skeletal muscle.

## 1 Introduction

Mitochondria are fundamental cellular components that underpin skeletal muscle function and, consequently, contribute to physical well-being and athletic performance (Joyner and Coyle, 2008; Nunnari and Suomalainen, 2012). Earlier studies focused mainly on changes in mitochondrial enzyme activity, such as citrate synthase (CS), as a biomarker of mitochondrial content in skeletal muscle (Larsen et al., 2015). Changes in mitochondrial respiratory function were presumed to be proportional to mitochondrial content (Hoppeler et al., 1987). However, subsequent studies reported decreased mitochondrial respiration without changes in mitochondrial enzyme activity in patients with type 2 diabetes (Mogensen et al., 2007; Phielix et al., 2008). Another study reported that, after a period of training, subjects with higher mitochondrial respiration resulted in greater exercise performance despite no significant increase in mitochondrial enzyme activity (Granata et al., 2016). These studies suggest that mitochondrial respiratory function can be dissociated from mitochondrial content and may be relevant to physical fitness and exercise performance. Thus, elucidating factors that alters mitochondrial respiratory function would contribute to establishing effective strategy for improving athletic performance and physical health.

Lactate is not merely an end product of glycolysis, but also a metabolic intermediate that serves as an oxidizable substrate and gluconeogenic precursor (Gladden, 2004; Brooks, 2009, 2018). In recent years, lactate has attracted considerable attention as a potential signaling molecule that induces physiological adaptations (Brooks, 2020; Brooks et al., 2021). Intriguingly, a previous study demonstrated that incubation of L6 cells with lactate increased the mitochondrial protein content (Hashimoto et al., 2007). Additionally, we previously reported that lactate administration enhanced mitochondrial enzyme activity in mouse skeletal muscle (Takahashi et al., 2019b; Takahashi et al., 2020b). Other researchers previously reported the improved mitochondrial respiration in skeletal muscle following high-intensity training (Granata et al., 2016), during which the circulating lactate level increases. These observations led us to hypothesize that lactate would possess a potential to alter mitochondrial respiratory function in skeletal muscle.

In this study, mitochondria isolated from the gastrocnemius muscle of mice administered with lactate for 4 weeks were suspended in four different media (pyruvate + malate, glutamate + malate, succinate + rotenone, and malate + octanoyl-l-carnitine) in the presence (state 3, maximal/ATP synthesis-coupled) or absence (state 2, basal/proton conductance) of ADP. Moreover, we evaluated mitochondrial enzyme activities and protein contents in the isolated mitochondria to better understand the mechanisms underlying lactate-induced mitochondrial functional adaptations.

## 2 Materials and Methods

### 2.1 Animals

Male ICR mice (8 weeks old; CLEA Japan, Tokyo, Japan) were used in this study. Mice were housed in an air-conditioned room at 22°C with a 12 h light-dark cycle (dark 7:00 a.m. to 7:00 p.m.). All mice were provided with standard chow (MF diet, Oriental Yeast, Tokyo, Japan) and water *ad libitum* during the experimental period. All experiments were approved by the Animal Experimental Committee of The University of Tokyo (approval number: 27–14).

### 2.2 Experimental Procedure

The animals were randomly divided into control (CON; *n* = 10) and lactate (LAC; *n* = 9) groups. They received intraperitoneal injections of phosphate-buffered saline (PBS) or sodium lactate (1 g/kg of body weight) 5 days a week for 4 weeks. The administration was performed at the same time of day (10:00 a.m.) when animals were active. We previously reported that the method of administration and volume of lactate increased blood lactate concentration to an upper physiological level (∼20 mM), and returned blood lactate to basal level 3 h after the injection (Kitaoka et al., 2016; Takahashi et al., 2019b). Twenty-4 hours after the last administration, the gastrocnemius muscles were taken and used for mitochondrial analysis.

### 2.3 Mitochondrial Isolation

Mitochondria were isolated by differential centrifugation as previously described (Tomiya et al., 2019; Wakabayashi et al., 2020). Briefly, freshly excised gastrocnemius muscle was immediately placed in ice-cold buffer (PBS, 10 mM EDTA, pH 7.4), minced with scissors, supplemented with 0.025% trypsin, and then incubated on ice for 5 min. After tissue suspensions were centrifuged at 200  × g for 5 min at 4°C, the supernatants were discarded. Tissue precipitate was homogenized using a Tenbroeck tissue grinder (Wheaton, NJ, United States ) with 10 strokes in a buffer (50 mM MOPS, 100 mM KCl, 1 mM EGTA, 5 mM MgSO_4_, and 2.0 g/L BSA, pH 7.1). The homogenates were centrifuged at 500  × g for 10 min at 4°C to collect supernatants containing mitochondria. The supernatants were re-centrifuged at 10,000 × g for 10 min at 4°C to obtain the mitochondrial pellets. The mitochondrial pellets were washed with buffer (50 mM MOPS, 100 mM KCl, 1 mM EGTA, and 5 mM MgSO_4_) and resuspended in buffer (105 mM potassium-MES, 10 mM Tris, 30 mM KCl, 10 mM KH_2_PO_4_, 5 mM MgCl_2_, 1 mM EGTA, and 2.5 g/L BSA, pH 7.2). The protein concentration was determined using the BCA protein assay (TaKaRa BIO INC., Shiga, Japan). For the respiratory function assays, the protein concentration of the mitochondrial solution was adjusted to 2.0 mg/ml.

### 2.4 Mitochondrial Oxygen Consumption Rate (OCR)

Mitochondrial OCR was measured as previously described, with minor modifications (Kitaoka et al., 2019; Tomiya et al., 2019; Tamura et al., 2020; Wakabayashi et al., 2020). Briefly, freshly isolated mitochondria (20 µg) were incubated in buffer (105 mM potassium-MES, 10 mM Tris, 30 mM KCl, 10 mM KH_2_PO_4_, 5 mM MgCl_2_, 1 mM EGTA, and 2.5 g/L BSA, pH 7.2). Mitochondrial respiration was initiated by addition of the respiration buffers described below. Mitochondrial OCR was measured in an oxygen-monitoring 96-well microplate (OP96C, PreScan Precision Sensing, Regensburg, Germany) using a Tecan Spark multimode plate reader (Spark 20M, Tecan, Männedorf, Switzerland) (excitation: 540 nm; emission: 650 nm). Relative fluorescence changes were measured per minute and normalized to CS activity. Data without normalization are shown in Supplementary Figure S2. We previously reported that the mitochondrial preparation protocol did not increase the OCR after adding 10 mM cytochrome c (Kitaoka et al., 2019; Tomiya et al., 2019; Wakabayashi et al., 2020), suggesting that integrity of the mitochondrial outer membrane was not disturbed.

### 2.5 Respiration Buffers

#### 2.5.1 Pyruvate + Malate

Pyruvate + malate-induced (complex I-driven) state 3 (PM3) and 2 (PM2) respiration was measured by the addition of a PM3 (5 mM potassium pyruvate, 1.5 mM malic acid, 2.5 mM ADP, 10 mM Tris, 30 mM KCl, 10 mM KH_2_PO_4_, 5 mM MgCl_2_, 1 mM EGTA, 2.5 g/L BSA, pH 7.2) and a PM2 (5 mM potassium pyruvate, 1.5 mM malic acid, 10 mM Tris, 30 mM KCl, 10 mM KH_2_PO_4_, 5 mM MgCl_2_, 1 mM EGTA, 2.5 g/L BSA, pH 7.2) buffer, respectively.

#### 2.5.2 Glutamate + Malate

Glutamate + malate-induced (complex I-driven) state 3 (GM3) and 2 (GM2) respiration was measured by the addition of a GM3 (10 mM glutamic acid, 1.5 mM malic acid, 2.5 mM ADP, 10 mM Tris, 30 mM KCl, 10 mM KH_2_PO_4_, 5 mM MgCl_2_, 1 mM EGTA, 2.5 g/L BSA, pH 7.2) and a GM2 (10 mM glutamic acid, 1.5 mM malic acid, 2.5 mM ADP, 10 mM Tris, 30 mM KCl, 10 mM KH_2_PO_4_, 5 mM MgCl_2_, 1 mM EGTA, 2.5 g/L BSA, pH 7.2) buffer, respectively.

#### 2.5.3 Succinate + Rotenone

Succinate + rotenone-induced (complex II-driven) state 3 (S3) and 2 (S2) respiration was measured by the addition of an S3 (10 mM sodium succinate, 1 mM rotenone, 2.5 mM ADP, 10 mM Tris, 30 mM KCl, 10 mM KH_2_PO_4_, 5 mM MgCl_2_, 1 mM EGTA, 2.5 g/L BSA, pH 7.2) and an S2 (10 mM sodium succinate, 1 mM rotenone, 10 mM Tris, 30 mM KCl, 10 mM KH_2_PO_4_, 5 mM MgCl_2_, 1 mM EGTA, 2.5 g/L BSA, pH 7.2) buffer, respectively

#### 2.5.4 Malate + Octanoyl-L-Carnitine

Malate + octanoyl-l-carnitine-induced state 3 (MOc3) and 2 (MOc2) respiration was measured by the addition of an MOc3 (10 mM octanoyl-l-carnitine, 1.5 mM malic acid, 2.5 mM ADP, 10 mM Tris, 30 mM KCl, 10 mM KH_2_PO_4_, 5 mM MgCl_2_, 1 mM EGTA, 2.5 g/L BSA, pH 7.2) and an MOc2 (10 mM octanoyl-l-carnitine, 1.5 mM malic acid, 10 mM Tris, 30 mM KCl, 10 mM KH_2_PO_4_, 5 mM MgCl_2_, 1 mM EGTA, 2.5 g/L BSA, pH 7.2) buffer, respectively.

### 2.6 Mitochondrial Enzyme Activity

Mitochondrial enzyme activity was measured as previously described, with minor modifications (Takahashi et al., 2019b; Wakabayashi et al., 2020). Electron transport chain enzyme activity data were normalized to CS activity. Data without normalization are shown in Supplementary Figure S3.

#### 2.6.1 CS

Mitochondrial aliquots were mixed with a reaction mixture (100 μM DTNB, 300 μM acetyl-CoA, and 50 μM oxaloacetate) in a 96-well plate. The changes in absorbance were determined at 412 nm/min. We confirmed that CS activity in the isolated mitochondria did not significantly differ between the two groups (CON: 1.00 ± 0.12; LAC: 0.96 ± 0.19, relative to CON) (Supplementary Figure S1A).

#### 2.6.2 *ß*-Hydroxyacyl-CoA Dehydrogenase (β-HAD)

Mitochondrial aliquots were mixed with a reaction mixture (1 M Tris, 5 mM EDTA, 450 µM NADH, and 100 µM acetoacetyl-CoA, pH 7.0) in a 96-well microplate. The absorbance changes at 340 nm/min were determined.

#### 2.6.3 Complex I

Mitochondrial aliquots were mixed with a reaction mixture (100 μM NADH, 60 μM ubiquinone, 300 μM KCN, and 3 mg/ml BSA) in a 96-well plate. The changes in absorbance were determined at 340 nm/min. Rotenone-sensitive enzyme activity (i.e., rotenone absence − rotenone presence) was regarded as complex I enzyme activity.

#### 2.6.4 Complex I + III

Mitochondrial aliquots were mixed with a reaction mixture (200 μM NADH, 50 μM cytochrome c, 300 μM KCN, and 1.0 mg/ml BSA) in a 96-well plate. The changes in absorbance were determined at 550 nm/min.

#### 2.6.5 Complex II

Mitochondrial aliquots were mixed with a reaction mixture [20 mM succinate, 0.015% (wt/vol) 2,6-dichlorophenolindophenol, 12.5 μM decylubiquinone, 300 μM KCN, and 1 mg/ml BSA] in a 96-well plate. The changes in absorbance were determined at 600 nm/min.

#### 2.6.6 Complex II + III

Mitochondrial aliquots were mixed with a reaction mixture (10 mM succinate, 50 μM cytochrome c, and 300 μM KCN) in a 96-well plate. The changes in absorbance were determined at 550 nm/min.

#### 2.6.7 Complex IV

Mitochondrial aliquots were mixed with a reaction mixture (50 μM cytochrome c reduced with dithiothreitol) in a 96-well plate. The changes in absorbance were determined at 550 nm/min.

### 2.7 Western Blotting

Mitochondrial pellets were suspended in ice-cold radioimmunoprecipitation assay buffer (25 mM Tris–HCl, pH 7.6, 150 mM NaCl, and 1% NP-40) and mixed with sodium dodecyl sulfate-polyacrylamide gel electrophoresis (SDS-PAGE) sample buffer (199–16,132, Fujifilm Wako Pure Chemical, Osaka, Japan). Equal amounts of protein were loaded onto SDS-PAGE gels (10–15%) and separated by electrophoresis. After proteins were transferred to polyvinylidene difluoride (PVDF) membranes, western blotting was performed as described previously (Takahashi et al., 2020a; Takahashi et al., 2020b). The primary and secondary antibodies used in this study are described below. Blots were scanned and quantified using a ChemiDoc XRS (Bio-Rad Laboratories, Hercules, CA, United States ) system and Quantity One software (version 4.5.2; Bio-Rad), respectively. The respiratory chain protein content was normalized to the CS content. Data without normalization are shown in Supplementary Figure S4. We confirmed that CS protein content in the isolated mitochondria did not significantly differ between the two groups (CON: 1.00 ± 0.08; LAC: 1.01 ± 0.13, relative to CON) (Supplementary Figure S1B).

### 2.8 Primary and Secondary Antibodies

The primary antibodies used in this study were as follows: MitoProfile Total OXPHOS Rodent WB Antibody Cocktail [NADH dehydrogenase (ubiquinone) 1β subcomplex 8 (NDUFB8), succinate dehydrogenase complex subunit B (SDHB), ubiquinol-cytochrome c reductase core protein II (UQCRC2), ATP synthase, H+ transporting, mitochondrial F1 complex, α-subunit (ATP5A), ab110413, Abcam], NADH dehydrogenase (ubiquinone) iron-sulfur protein 4 (NDUFS4; ab139178, Abcam), cytochrome c oxidase subunit IV (COX IV; ab14744, Abcam), and CS (ab129095; Abcam). The following secondary antibodies were used in the present study: rabbit anti-goat IgG (H&L) (#A102PT; American Qualex, San Clemente, CA, United States ) and mouse anti-goat IgG (H&L) (#A106PU; American Qualex).

### 2.9 Mitochondrial Supercomplex Assembly Assay

Mitochondrial supercomplex assembly was evaluated by two-dimensional PAGE, as described previously (Wakabayashi et al., 2020). Mitochondrial specimens (50 µg of protein) were suspended in 5% digitonin [digitonin/protein ratio: 8 (g/g); BN 2006, Thermo Fisher Scientific] and NativePAGE™ 4X Sample Buffer (BN20032, Thermo Fisher Scientific). After incubation on ice for 30 min, the suspensions were centrifuged at 20,000 × g for 15 min at 4°C. The supernatants were mixed with NativePAGE™ 5% G-250 Sample Additive (BN 2004, Thermo Fisher Scientific). Samples were loaded on 4–15% gradient gels (4,561,096, Bio-Rad) and were separated by blue native PAGE (BN-PAGE) using native PAGE buffer (BN 2001, Thermo Fisher Scientific). Proteins were then separated by SDS-PAGE and transferred to PVDF membranes. Western blotting was performed using anti-oxidative phosphorylation antibodies (MitoProfile Total OXPHOS Rodent WB Antibody Cocktail; ab110413, Abcam), as described above.

### 2.10 Statistical Analysis

All data are expressed as mean ± standard deviation (SD). Comparisons between two groups were made using the unpaired Student’s t-test. Correlations between two variables were studied using least-squares linear regression followed by the Pearson’s correlation coefficient test. All statistical analyses were performed using the GraphPad Prism software (Ver. 9.0, Macintosh, GraphPad Software, La Jolla, CA). Statistical significance was defined as *p* < 0.05. To minimize type II errors, all the results within the range 0.05 ≤ *p* ≤ 0.10 are shown.

## 3 Results

**FIGURE 1:**
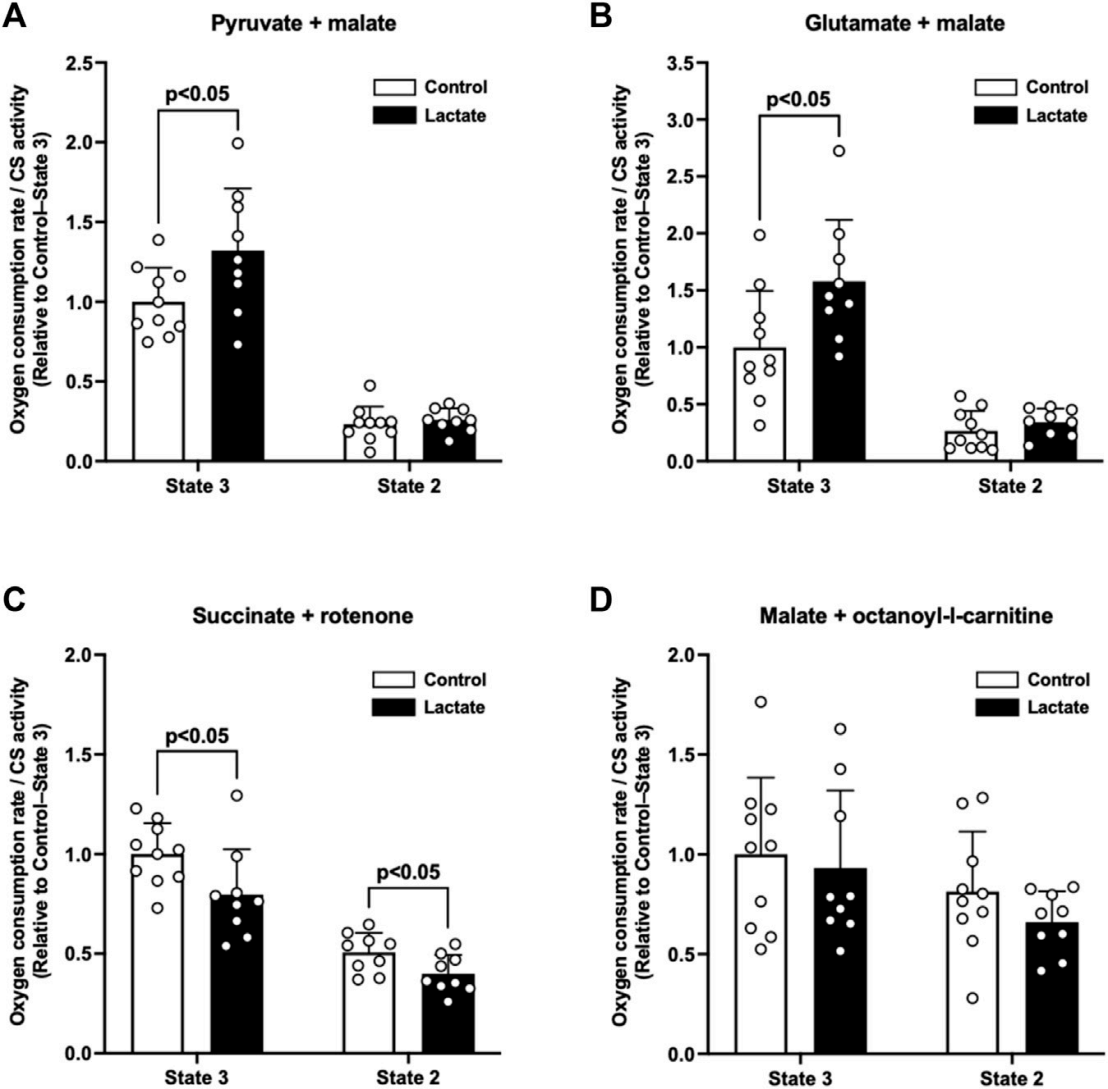
Oxygen consumption rate (OCR) in isolated mitochondria. **(A)** Pyruvate + malate-induced (complex I-driven) OCR. **(B)** Glutamate + malate-induced (complex I-driven) OCR. **(C)** Succinate + rotenone-induced (complex II-driven) OCR. **(D)** Malate + octanoyl-l-carnitine-induced OCR. Data are expressed as mean ± SD and are normalized to citrate synthase (CS) activity. Control group: *n* = 10, Lactate group: *n* = 9. Unpaired Student’s t-test was used for statistical evaluation.

To elucidate whether lactate administration enhances mitochondrial respiratory function, we measured the mitochondrial OCR under state 3 and 2 conditions. The LAC group exhibited significantly higher PM3 (*p* < 0.05; Figure 1A) and GM3 (*p* < 0.05; Figure 1B) than those of the CON group. In contrast to the complex I-driven OCR, S3 and S2 were significantly lower in the LAC group than those in the CON group (*p* < 0.05; Figure 1C). No differences were observed between the two groups in PM2 (Figure 1A), GM2 (Figure 1B), MOc3, and MOc2 (Figure 1D). Together, these observations suggest that lactate alters mitochondrial respiratory function.

**FIGURE 2:**
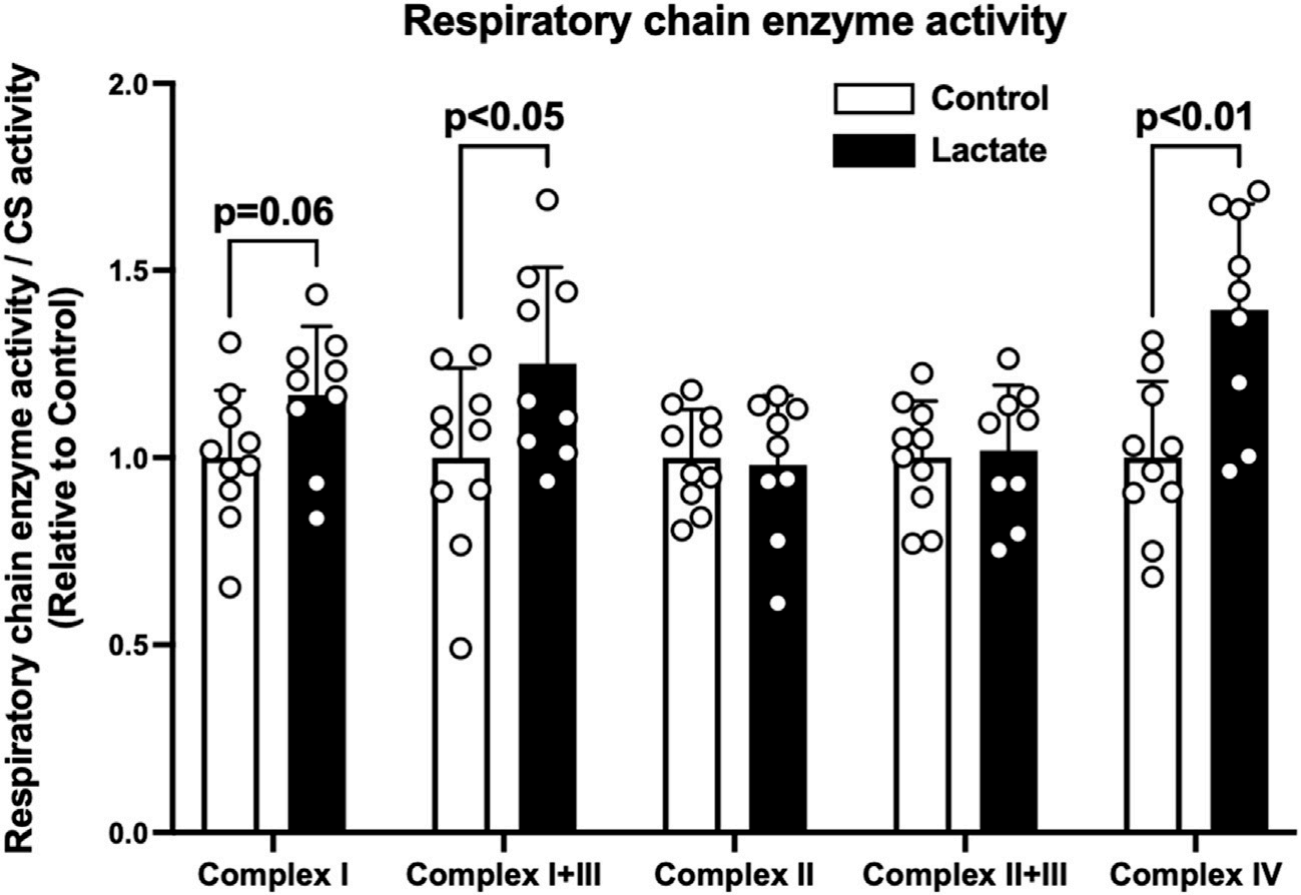
Mitochondrial respiratory chain enzyme activity. Data are expressed as mean ± SD and are normalized to citrate synthase (CS) activity. Control group: *n* = 10, Lactate group: *n* = 9. Unpaired Student’s t-test was used for statistical evaluation.

**FIGURE 3:**
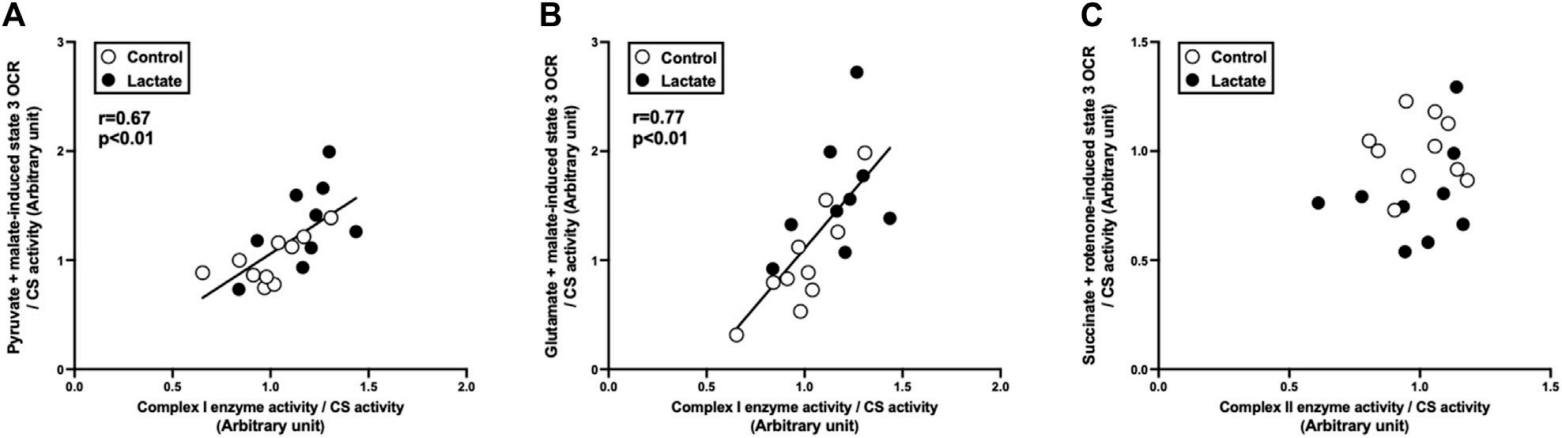
Correlation between state 3 oxygen consumption rate (OCR) and respiratory chain enzyme activity. **(A)** pyruvate + malate-induced (complex I-driven) state 3 OCR against complex I enzyme activity. **(B)** glutamate + malate-induced (complex I-driven) state 3 OCR against complex I enzyme activity. **(C)** succinate + rotenone-induced (complex II-driven) state 3 OCR against complex II enzyme activity. Data are expressed as mean ± SD. Control group: *n* = 10, Lactate group: *n* = 9. The correlation was studied using least-squares linear regressions, followed by the Pearson’s correlation coefficient test.

To clarify the mechanisms underlying the changes in mitochondrial respiration, we evaluated enzymatic activities of the electron transport chain (complexes I, I + III, II, II + III, and IV). Complex I activity tended to be higher in the LAC group than that in the CON group (*p* = 0.06; Figure 2). Additionally, the activities of complexes I + III and IV were significantly higher in the LAC group than those in the CON group (*p* < 0.05; Figure 2). No significant differences were observed in the activities of complexes II and II + III (Figure 2). Moreover, complex I activity positively correlated with PM3 (r = 0.67, *p* < 0.01; Figure 3A) and GM3 (r = 0.77, *p* < 0.01; Figure 3B). No significant correlation between the activity of complex II and S3 was found (Figure 3C). Overall, it is likely that the increase in PM3 and GM3 respiration results from the enhanced complex I capacity. However, decline in S2and S3 respiration are not likely to be explained by complex II capacity.

**FIGURE 4:**
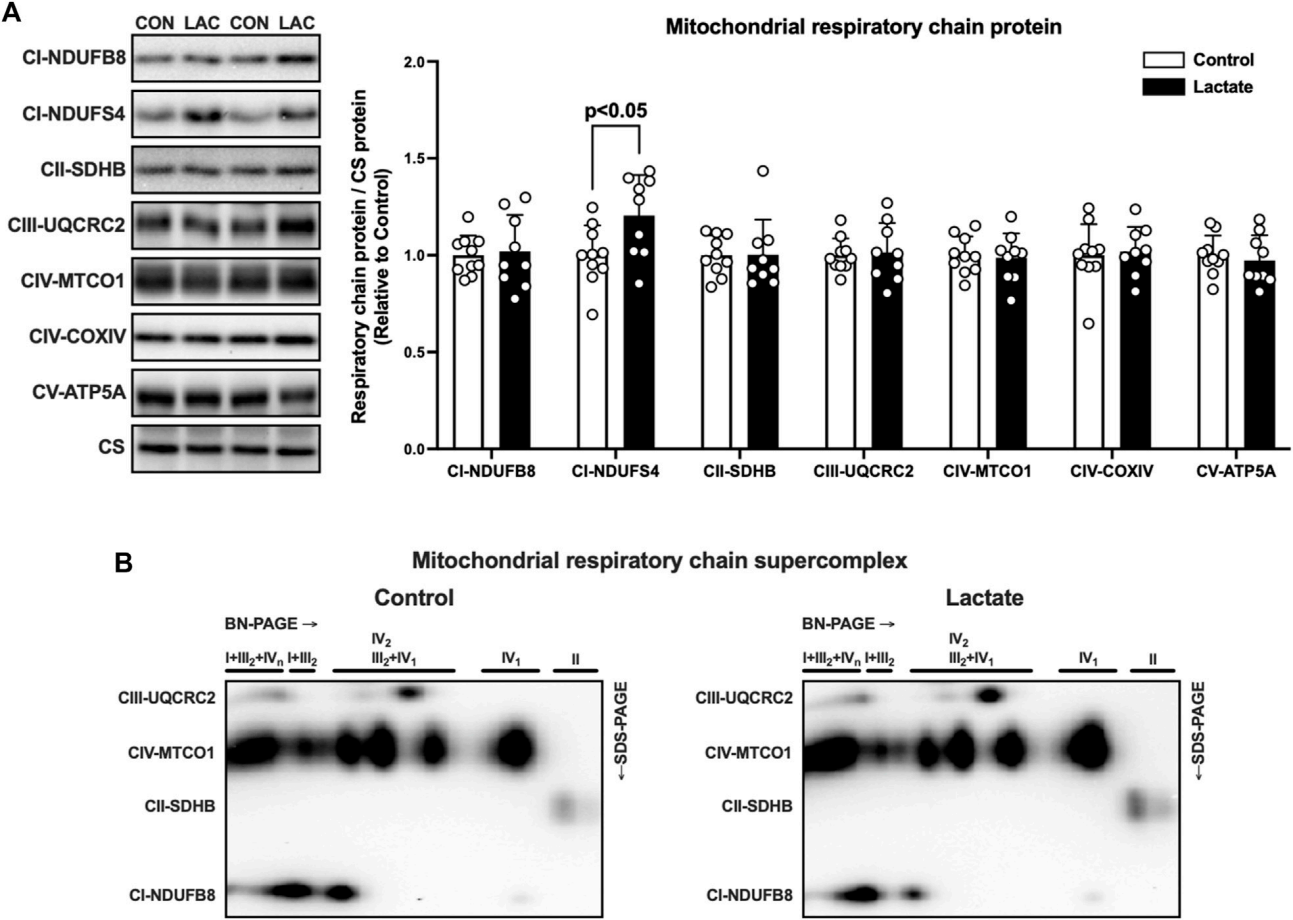
The protein content of respiratory chain components **(A)** and supercomplex assembly **(B)** in isolated mitochondria. Data are expressed as mean ± SD and are normalized to citrate synthase (CS) protein. Control group: *n* = 10, Lactate group: *n* = 9. Unpaired Student’s t-test was used for statistical evaluation.

To further explore the mechanisms whereby mitochondrial respiration and electron transport chain enzyme activity were altered, we assessed the mitochondrial protein content and supercomplex assembly. First, we measured the protein content of the electron transport chain components. The protein content of NDUFS4, a subunit of complex I, was significantly higher in the LAC group than that in the CON group (Figure 4A). No significant differences were observed in protein content of NDUFB8, SDHB, UQCRC2, MTCO1, COXIV, and ATP5A (Figure 4A). These observations suggest that the increased NDUFS4 protein content may contributed to the enhanced complex I enzyme activity. Emerging evidence suggests that the mitochondrial supercomplex organization contributes to mitochondrial respiration (Ikeda et al., 2013; Greggio et al., 2017). Next, we evaluated mitochondrial supercomplex assembly using two-dimensional PAGE (BN-PAGE followed by SDS-PAGE). No differences in supercomplex assembly were observed between the CON and LAC groups (Figure 4B), suggesting that lactate administration changed mitochondrial respiration and electron transport enzyme activity without altering mitochondrial supercomplex assembly.

## 4 Discussion

### 4.1 Key Findings and Perspectives

Research has shown that not only mitochondrial content but also mitochondrial respiratory function is associated with exercise performance and metabolic disorders (Phielix et al., 2008; Granata et al., 2016). Although we and others previously reported that lactate increases mitochondrial content biomarkers, such as enzyme activity and protein content, in L6 cells (Hashimoto et al., 2007) and mouse skeletal muscle (Takahashi et al., 2019b; Takahashi et al., 2020b), whether lactate alters mitochondrial respiratory function remains to be elucidated. In the current study, we showed that 4 weeks of lactate administration enhanced PM3 and GM3 respiration, and decreased S3 and S2 respiration. These novel findings suggest that lactate changes mitochondrial respiratory function in skeletal muscle.

Although mitochondrial respiratory capacity is typically proportional to mitochondrial content (Hoppeler, Hudlicka and Uhlmann 1987; Jacobs et al., 2013), a dissociation between these two variables has been observed in mice (Rowe et al., 2013) and humans (Jacobs and Lundby 2013; Montero et al., 2015; Granata et al., 2016; Meinild Lundby et al., 2018). A previous study reported that sprint interval training, but not traditional endurance training, enhanced mitochondria-specific respiration in skeletal muscle without changes in mitochondrial content (Granata et al., 2016), although most studies have reported the increased mitochondrial content after high-intensity training (Gibala et al., 2006; Burgomaster et al., 2008; Jacobs et al., 2013). In contrast, traditional endurance training decreased intrinsic mitochondrial respiration in skeletal muscle, albeit, with an increase in mitochondrial content (Montero et al., 2015; Meinild Lundby et al., 2018). Based on these observations, it is believed that high training intensity contributes to the improvement of mitochondrial respiration rather than mitochondrial content (Bishop, Granata and Eynon 2014; Granata, Jamnick and Bishop 2018; Bishop et al., 2019). We have previously reported that the method of administration and volume of lactate used in this study increased the blood lactate concentration similar to a level that could be reached during high-intensity exercise (∼20 mM) (Kitaoka et al., 2016; Takahashi et al., 2019b). In the current study, we observed that 4 weeks of lactate administration enhanced PM3 and GM3 respiration. Taken together, improved mitochondrial respiration in skeletal muscle following high-intensity training may result, in part, from lactate production during exercise.

### 4.2 Substrate-dependent Mitochondrial Respiration

In the present study, we evaluated substrate- and complex-dependent mitochondrial respiratory function using different substrate combinations. Substrate combinations of pyruvate + malate and glutamate + malate stimulate dehydrogenases to produce NADH, which provides electrons for complex I. Flux through pyruvate dehydrogenase (PDH), a gateway enzyme for the entry of pyruvate into the tricarboxylic acid cycle, has been suggested to be a rate-limiting factor for pyruvate oxidation in the presence of malate (Bookelman et al., 1978; Lemieux, Tardif and Blier 2010). In the current study, complex I-driven state 3 respiration, irrespective of substrate combination (pyruvate + malate or glutamate + malate), was positively correlated with complex I enzyme activity. These observations suggest that, when mitochondrial respiration is maximally activated with adequate complex I substrates and ADP, NADH consumption by complex I appears to be a key step for mitochondrial oxygen consumption and subsequent ATP production.

In line with our previous observation that *Ndufs4* mRNA level in mouse skeletal muscle increased 3 h after a single lactate administration (Kitaoka et al., 2016), we presently found that NDUFS4 protein level in isolate mitochondria increased after 4 weeks of lactate administration. In NDUFS4 deficiency, complex I fails to assemble properly or is unstable (Petruzzella et al., 2001), resulting in reduced complex I activity and complex I-driven respiration (Kruse et al., 2008; Ingraham et al., 2009). This suggests that NDUFS4 plays a key role in complex I function. Thus, the present observations of elevated PM3 and GM3 respiration associated with complex I enzyme activity may be explained, at least in part, by an increase in NDUFS4 protein levels.

Contrary to complex I-driven state 3 respiration, lactate administration diminished S3 and S2 respiration without changes in complex II enzyme activity or component protein (SDHB) level. These observations suggest that FADH_2_ production and consumption by complex II (SDH) do not explain the decline in S3 and S2 respiration. One possible explanation is inhibition of the dicarboxylate carrier that accounts for the uptake of succinate by isolated mitochondria. Previous studies have reported that the rate of uptake in mitochondria as catalyzed by the dicarboxylate carrier of dicarboxylates was inhibited in the presence of other intermediate metabolites (van Dam and Tsou 1968; Palmieri et al., 1971). Future studies are required to clarify whether lactate administration changes mitochondrial intermediary metabolism, which impairs mitochondrial succinate uptake and succinate-fueled respiration.

The observation of increased PM3 and GM3 respiration with decreased S3 respiration indicates that possible metabolic remodeling in favor of complex I substrates relative to complex II substrates occurred. In the skeletal muscle of streptozotocin-induced type 1 diabetic rats, complex I-driven respiration has increased despite no difference in complex I + II-driven respiration, where the substrate control ratio for succinate and complex II protein content are reduced (Larsen et al., 2015). Other studies have shown that electron flow through complex II increases in complex I-deficient mice (Alam et al., 2015; Terburgh et al., 2019). These observations suggest that complexes I and II reciprocally compensate for deteriorated complex functions. Importantly, another study reported that resistance training increased complex I-driven respiration together with complex I protein abundance, while the substrate control ratio for succinate was reduced even though complex II protein levels remained unchanged (Porter et al., 2015). This suggests that augmented electron transfer through complex I may result in diminished complex II electron transfer. Thus, the observation of reduced S3 respiration is likely due to improved complex I function rather than a decline in complex II abundance or function.

NADH and FADH_2_ generated in the course of fatty acid oxidation are substrates for complex I and the electron-transferring flavoprotein complex. In the present study, although we observed a tendency to increase complex I enzyme activity and a significant increase in complex I + III enzyme activity after lactate administration, neither MOc3 nor MOc2 respiration was changed. While both pyruvate and fatty acids are converted to acetyl-CoA and NADH, the maximal flux through PDH to produce these metabolites from pyruvate is much higher than the reaction of fatty acid oxidation (Petrick and Holloway 2019). These observations suggest that NADH production through fatty acid oxidation did not reach the maximal capacity of complex I to consume NADH, and that reactions other than NADH consumption are key events for fatty acid-induced mitochondrial respiration. We previously reported that high-intensity training enhanced palmitate-induced oxygen consumption with the increased activity of *ß*-HAD, through which NADH is yielded, in the isolated mitochondria of rat skeletal muscle (Hoshino et al., 2013). In the present study, *ß*-HAD activity did not change after lactate administration (CON: 1.00 ± 0.16; LAC: 0.99 ± 0.10, relative to CON) (Supplementary Figure S1C). Collectively, it appears that increasing NADH production through *ß*-HAD reaction is likely to be a key step for fatty acid-induced mitochondrial respiration.

### 4.3 Speculations as to Why Lactate Administration Enhanced Complex I Capacity

The conversion of lactate to pyruvate and subsequent pyruvate oxidation in the tricarboxylic acid cycle yield NADH, which is then consumed by complex I. Previous studies have reported an elevated lactate concentration in the circulation and tissues of NDUFS4 deficient mice (Kruse et al., 2008; Ingraham et al., 2009; Jin et al., 2014; Saito et al., 2019). Additionally, humans with a mutation of NDUFS4 genes, including patients with Leigh syndrome, have repeatedly shown an increased blood lactate level (Budde et al., 2000; Loeffen et al., 2000; Petruzzella et al., 2001; Iuso et al., 2006; Assouline et al., 2012). Without proper function of complex I due in part to loss of NDUFS4, pyruvate cannot be oxidized in mitochondria, but instead, converted to lactate to regenerate NAD+ (Ingraham et al., 2009). These suggest that decline in complex I capacity cause lactate accumulation; on the contrary, increasing complex I capacity enhances lactate oxidation. Therefore, it is speculated that lactate enhances complex I capacity, presumably enabling the mitochondria to preferentially oxidize lactate in the condition that abundant lactate is available. In support of this notion we previously observed that, after 3 weeks of lactate administration with endurance training, lactate removal from the blood circulation in mice was enhanced to a greater extent than with endurance training alone (Takahashi et al., 2019b).

### 4.4 Potential Mechanisms and Future Prospective

The molecular mechanisms whereby lactate administration induces mitochondrial adaptation remain unclear. Growing evidence has shown that lactate regulates cell signaling and adaptation (Brooks 2020; Brooks et al., 2021). Previous studies have reported that lactate activates cell signaling by binding to hydroxycarboxylic acid receptor 1 (Ahmed et al., 2010; Ohno et al., 2018). Another study reported that lactate facilitated adipose secretion of TGF-β, which subsequently enhanced mRNA expression of *peroxisome proliferator-activated receptor γ coactivator 1-α* (*Pgc-1α*), a master regulator of mitochondrial adaptation (Wu et al., 1999), in mouse skeletal muscles (Takahashi et al., 2019a), suggesting that inter-organ communication activated by lactate possibly contributed to the current findings. Other researchers have identified a lactate-responsive form of PGC1 (LRPGC1), which has been reported to enhance lactate consumption by enhancing estrogen-related receptor γ-mediated transcription in mouse hepatic and renal cells (Tanida, Matsuda and Tanaka 2020). Furthermore, a previous study demonstrated metabolic regulation of gene expression by histone lactylation in cancer cells (Zhang et al., 2019). Lastly, it should be noted that exposure to high lactate levels can change the catabolic fate of pyruvate in skeletal muscle and other cells. Because of mass action restrictions, relatively little, if any, pyruvate is reduced to lactate, which instead needs to be oxidized by mitochondria. This relatively high substrate pressure can cause a reduced mitochondrial environment with increased NADH/NAD^+^ and ATP/ADP ratios, increased protonmotive force and, related, increased likelihood of reactive oxygen species generation, which is considered to trigger gene expressions, leading to enhanced oxidative activity and capacity (Hashimoto et al., 2007; Brooks 2020). Whether these previous findings explain the current observations of altered mitochondrial function should be clarified in future studies.

## 5 Conclusion

The present study examined whether lactate administration alters mitochondrial respiratory function in skeletal muscle. Using isolated mitochondria, we demonstrated that lactate administration increased pyruvate + malate- and glutamate + malate-induced (complex I-driven) mitochondrial respiration, with a tendency to increase complex I enzyme activity and a significant increase in complex I component protein. Additionally, lactate administration decreased succinate + rotenone-induced (complex II-driven) mitochondrial respiration, despite no changes in complex II enzyme activity or component proteins. These findings suggest that lactate alters mitochondrial respiratory function, and that exercise training-induced increases in mitochondrial respiration may result, in part, from lactate production during exercise.

## Data Availability

The raw data supporting the conclusion of this article will be made available by the authors, without undue reservation.
