# Supplementary material for: Effects of Lactate Administration on Mitochondrial Respiratory Function in Mouse Skeletal Muscle
Source: Front Physiol. 2022 Jun 30;13:920034. doi: 10.3389/fphys.2022.920034 (PMC9280083; doi:10.3389/fphys.2022.920034)
Supplement: Supplementary file 1 [file DataSheet1.PDF]

Supplementary Material

Supplementary Figures

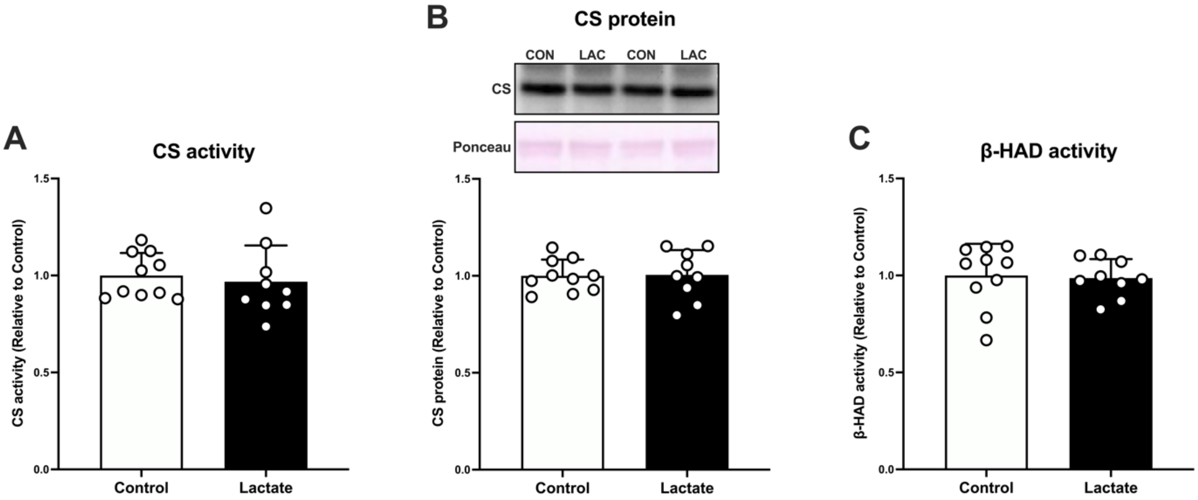

Supplementary Figure 1. Mitochondrial enzyme and protein levels in isolated mitochondria. A: Citrate synthase (CS) activity. B. Citrate synthase (CS) protein. C: β-hydroxyacyl-CoA dehydrogenase (β-HAD) activity. Data are expressed as mean ± SD. Control group: n = 10, Lactate group: n= 9. Unpaired Student's t-test was used for statistical evaluation.

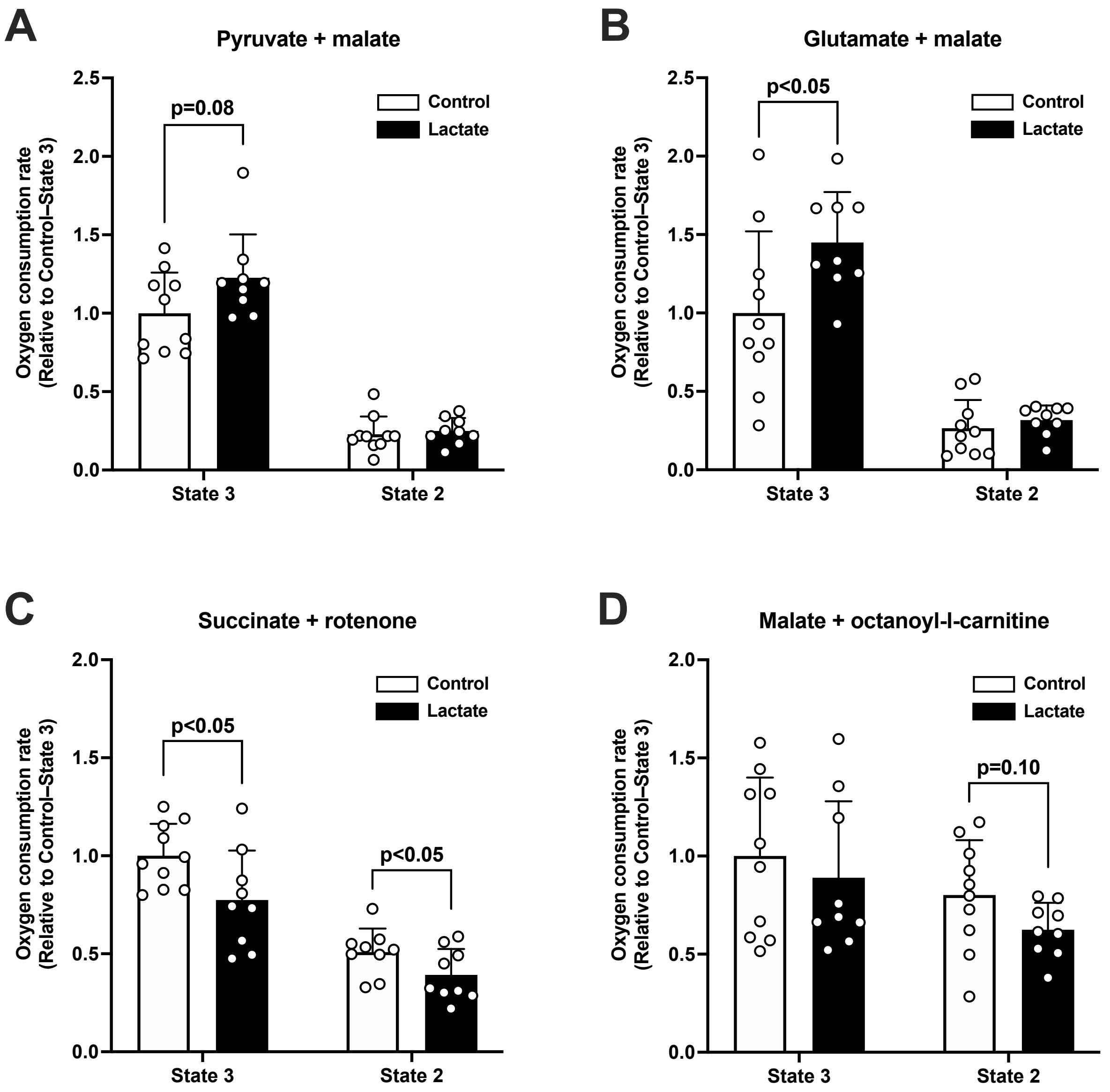

Supplementary Figure 2. Oxygen consumption rate (OCR) in isolated mitochondria. A: Pyruvate + malate-induced (complex I-driven) OCR. B: Glutamate + malate-induced (complex I-driven) OCR. C: Succinate + rotenone-induced (complex II-driven) OCR. D: Malate + octanoyl-l-carnitine-induced OCR. Data are expressed as mean ± SD. Control group: n = 10, Lactate group: n= 9. Unpaired Student's t-test was used for statistical evaluation.

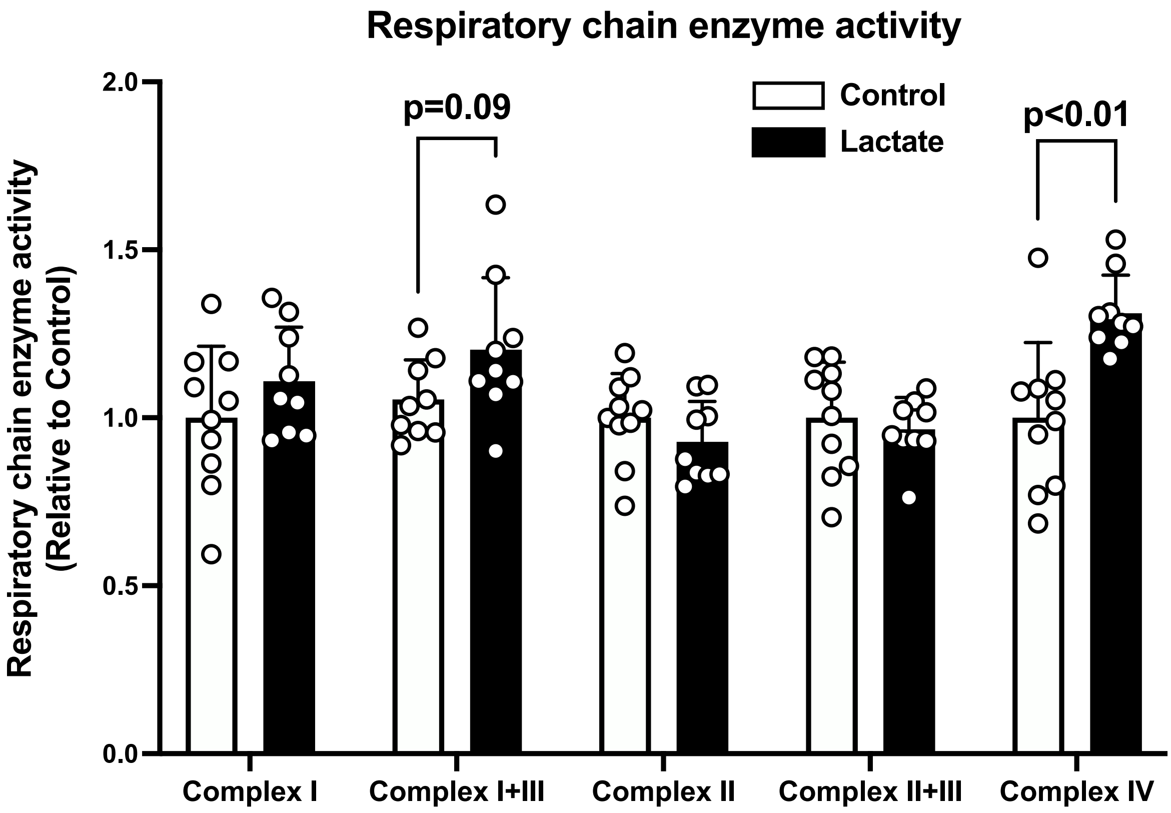

Supplementary Figure 3. Mitochondrial respiratory chain enzyme activity. Data are expressed as mean ± SD. Control group: n = 10, Lactate group: n= 9. Unpaired Student's t-test was used for statistical evaluation.

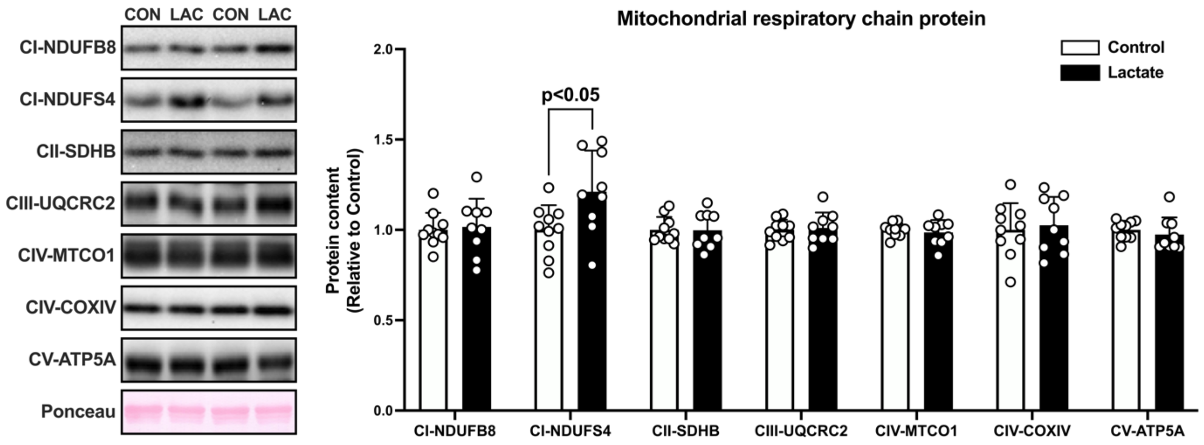

Supplementary Figure 4. The protein content of respiratory chain components in isolated mitochondria. Data are expressed as mean ± SD. Control group: n = 10, Lactate group: n= 9. Unpaired Student's t-test was used for statistical evaluation.
